# Supplementary material for: Mindful self-focus–an interaction affecting Theory of Mind?
Source: PLoS One. 2023 Feb 2;18(2):e0279544. doi: 10.1371/journal.pone.0279544 (PMC9894420; doi:10.1371/journal.pone.0279544)
Supplement: S1 File — (ZIP) [file pone.0279544.s001.zip › READ ME.rtf]

How to reproduce the results:1. Open and close the R Project file  “MindYourOwn.Rproj2. Open Processing and Analysis > Command Files > “MindYourOwn.Rmd3. Make sure the packages are installed. If you have to install some packages first, close and open the .Rmd after installing once more before running the script.4. Run the script.Note: Some non-essential code chunks (e.g. to export figures) has been turned into comments but can be uncommented to run if required.
