# Supplementary material for: Mindful self-focus–an interaction affecting Theory of Mind?
Source: PLoS One. 2023 Feb 2;18(2):e0279544. doi: 10.1371/journal.pone.0279544 (PMC9894420; doi:10.1371/journal.pone.0279544)
Supplement: S1 File — (ZIP) [file pone.0279544.s001.zip › mindful_selffocus_and_ToM_mainDoc.docx]

**Mindful Self-Focus–An Interaction Affecting Theory of Mind (ToM)?**

**Abstract**

Is thinking about oneself helpful or harmful for understanding other people? The answer might depend on how a person thinks about themself. We hypothesize that the relationship between self-focus and Theory of Mind (ToM) is moderated by mindfulness. We evaluate our hypothesis with a large cross-sectional dataset (*N* = 543) of native and non-native German and English speakers using OLS and MM-estimated robust multiple regression analysis. We found a small but robust self-focus × mindfulness interaction effect on ToM so that there was a significant positive relation between self-focus and ToM for more mindful individuals and no significant relation for less mindful individuals. The findings support our hypothesis that mindfulness moderates the relationship between self-focus and ToM performance. We discuss the limitations and differences between the present study and previous findings.

*Keywords:* self-focus, Theory of Mind, mindfulness, social cognition, egocentrism

*Data Availability Statement:* Data and analysis script are publicly available through the Open Science Framework here: ([anonymized link](https://osf.io/yneu7/?view_only=b918e2a6f3754411a0079d4f5f1db7e7))

**Mindful Self-Focus–An Interaction Affecting Theory of Mind (ToM)?**

Is thinking about oneself helpful or harmful for understanding other people? *Self-focus* is the tendency to attend to one’s own thoughts, feelings, and intentions (Exner, 1973; Ingram, 1990), while *Theory of Mind* (ToM) is the ability to infer these in other people (Frith & Frith, 2005; Preckel, Kanske, & Singer, 2018). Intuitively, thinking about oneself should biases inferences about other people in an egocentric manner (Fenigstein & Abrams, 1993). *Objective self-awareness theory*, however, posits that it actually reduces this bias (Duval & Wicklung, 1972; Hass & Eisenstadt, 1990; Silvia & Duval, 2001). A third possibility is that self-focus can be helpful or harmful depending on *how* one thinks about oneself.

This third option is derived from the *meta-construct model* (Ingram, 1990; Panayiotou, 2004). Self-focus is part of normal psychological functioning, yet historically it has often been considered regarding excessive self-focus associated with negative affect, anxiety, depression, and virtually every mental disorder (Harvey, Watkins, Mansell, & Shafran, 2004; Mor & Winquist, 2002; Woodruff-Borden, Brothers, & Lister, 2001). Herein, Ingram suggested that one should distinguish between the pervasive *process* of self-focus and its specific *content* or *quality* when evaluating its role.

Here, we explore this idea by investigating whether the relationship between *self-focus* and *ToM* performance is moderated by *mindfulness*. *Mindfulness* is the tendency to be conscious of what is going on in the present moment within oneself and in one’s surroundings including other people (Bishop et al., 2004; Brown & Ryan, 2003). Previous research has shown that reflective and mindful self-focus has many benefits for psychological functioning making mindfulness a likely moderator for our hypothesis (Huffziger & Kuehner, 2009; McKie, Askew, & Dudley, 2017; Sauer & Baer, 2012; Trapnell & Campbell, 1999; Watkins, 2008; Watkins & Teasdale, 2004).

**The Outcome: ToM**

In real life, the ability to reason about other people’s mental states requires the consideration of past, present, general, and occasion-specific information about people and social situations (Achim, Guitton, Jackson, Boutin, & Monetta, 2013). Additionally, one’s own mental state regarding some context may provide valuable insight into other people’s perspectives. Often, what is shared already explains quite a lot (Furr, 2008). However, one can also be mistaken to project one’s mental state onto other people or believe they are likeminded, that is *egocentric bias* and *false consensus belief*, respectively (Preckel et al., 2018; Ross, Greene, & House, 1977; Ross & Sicoly, 1979). Thus, the crux for successful ToM is appropriately differentiating between oneself and another person, that is *self–other distinction* (Steinbeis, 2016).

Notably, bias and accuracy are not necessarily opposites; in the right circumstances, bias can facilitate accuracy because bias allows for robust predictions under uncertainty (Gigerenzer & Brighton, 2009; Gigerenzer & Gaissmaier, 2010). In other words, a person can be right for the “wrong” reason like when grounding inferences about other people’s mental states in their own mental state rather than information about the other person. This is important because most ToM tasks measure either accuracy or bias but not both. Thus, they actually cannot answer whether in real life more egocentric participants will be less accurate or *vice versa.* Previous research on self-focus and ToM has mostly employed measures of egocentric bias, while here an accuracy measure was used.

**The Predictor: Self-focus**

As mentioned, the tendency to focus on one’s own mental states has often been considered from a psychopathological perspective. Much research differentiated between private and public, positive and negative, or reflective and ruminative self-focus (Mor & Winquist, 2002) reinforcing the idea that the role of self-focus depends on its quality (Ingram, 1990; Panayiotou, 2004).

Nevertheless, most research focuses on the main effects of some kind of self-focus. For example, some studies have suggested a negative effect (Chiou & Lee, 2013; Fenigstein & Abrams, 1993), and others have suggested a positive effect of self-focus on ToM (Abbate, Boca, & Gendolla, 2016; Gendolla & Wicklund, 2009). With tasks like (a) writing an E on one’s forehead, (b) judging how a third party would interpret a sentence the participant knows to be meant sarcastically, or (c) estimating how many peers share one’s preferences, these studies examine the role of self-focus for egocentric bias but not for ToM accuracy.

We found only one study that employed a ToM accuracy measure, specifically *emotion recognition* (Dinulescu et al., 2020). Therein, accuracy was based on the comparison of participant ratings of the emotions conveyed in different video clips and the actors’ self-ratings of their enacted emotions. However, the study did not concern self-focus but the related construct of *self-referential processing* which concerns processing of information that has been related to oneself (Rogers, Kuiper, & Kirker, 1977). They found that participants who better retrieved self-related information were also more accurate in judging other’s emotions.

On grounds of research on objective self-awareness theory (Duval & Wicklung, 1972; Hass & Eisenstadt, 1990; Stephenson & Wicklund, 1983), self-focus seems to be positively related to ToM. Arguably, it facilitates taking a third-person perspective on one’s own perspective. In other words, it should help with appropriate self–other distinction to account for otherwise misplaced egocentric bias. This leads to our first cautious hypothesis:

*(H1) If there is a main effect of self-focus on ToM at all, it is probably positive.*

**The Moderator: Mindfulness**

Being aware of the present moment is relevant to both inward self-focus and outward ToM. Quite generally, research suggests a positive relation of mindfulness and most social cognitive abilities (Campos et al., 2019; Fuochi & Voci, 2020). Disputed is mainly the underlying mechanism. Suggestions include among others that mindfulness improves ToM (a) by increasing self-knowledge or self-compassion (Block-Lerner, Adair, Plumb, Rhatigan, & Orsillo, 2007; Bornemann, Herbert, Mehling, & Singer, 2015; Kingsbury, 2009), (b) by simply motivating a person to engage more in ToM (Doesum, Van Lange, & Van Lange, 2013), or (c) decreasing egocentric biases directly or indirectly through changes in affect (Golubickis, Tan, Falben, & Macrae, 2016; Raglan & Schulkin, 2014; Weger, Hooper, Meier, & Hopthrow, 2012). Thus, we hypothesize:

*(H2) There is a positive main effect of mindfulness on ToM.*

Turning to mindfulness as a moderator, a range of distinct adaptive properties have been discerned for reflective and mindful self-focus on mood and psychological functioning (Huffziger & Kuehner, 2009; Sauer & Baer, 2012; Trapnell & Campbell, 1999; E. Watkins & Teasdale, 2004). For example, it has recently been shown that paranoid thinking is maintained by ruminative self-focus but reduced by mindful self-focus (McKie et al., 2017).

Regarding ToM specifically, however, evidence is sparse. We found only one study that tested how the effect of self-focus on ToM was moderated but therein, the moderator was negative affect (Yang, Yang, & Chiou, 2010). After inducing states of shameful, guilty, or neutral self-focus, the researchers asked participants to judge how sarcastic an uninformed third person would interpret a message praising a poor restaurant experience. Ashamed individuals expected a more sarcastic interpretation and guilt-ridden individuals expected a less sarcastic interpretation than individuals in the neutral condition. This illustrates that the direction of a person’s egocentric bias can change depending on the (affective) quality of self-focus. Taken together with Ingram’s (1990) suggestion, this motivates our central hypothesis:

*(H3) Mindfulness positively moderates the relation of self-focus and ToM.*

**Control Variables**

***Negative Affect***

The tendency or state of experiencing negatively valanced feelings (Diener & Emmons, 1984; Watson, Clark, & Tellegen, 1988) has come up multiple times. Negative affect is a broad construct comprising different feelings that in themselves serve widely different socio-psychological functions (Bodenhausen, Sheppard, & Kramer, 1994; Schimmack, Oishi, Diener, & Suh, 2016). Different affective states can play different roles for both thinking style and thought content depending on the context and the object of affect attributions (Baanders, 1993; Clore, Schiller, & Shaked, 2018; Forgas, 2002; Green & Sedikides, 2016).

Thus, it should not be surprising that relation of negative affect to self-focus and ToM is not clear cut (Huffziger & Kuehner, 2009; Ingram, 1990; Mor & Winquist, 2002; Sauer & Baer, 2012; Trapnell & Campbell, 1999; E. R. Watkins, 2008; E. Watkins & Teasdale, 2004; Woodruff-Borden et al., 2001). Case in point are the study on the role of shameful and guilt-ridden self-focus for egocentric bias (Yang et al., 2010) and a similar study suggesting that states of anxiety and surprise, but not anger or disgust drive individuals to rely more on their own perspective (Todd, Forstmann, Burgmer, Brooks, & Galinsky, 2015). In contrast, the relation between negative affect and mindfulness clearly seems to be negative with an *r* = -.39 (Schroevers & Brandsma, 2010).

We consider negative affect an important control variable, because it seems to be related to both self-focus and ToM–though at the domain level the direction remains unclear.

***Other Influential Variables***

There are several additional variables we consider: age, years of education, gender, participation language, language nativity, and ToM task attention. Although we do not expect either of them to drastically change the hypothesized relations, they are likely candidates to explain some of the variance and provide some context for the interpretation of the effects of interest (Spector & Brannick, 2010): We expect ToM performance to be negatively related to age (Henry, Phillips, Ruffman, & Bailey, 2013) but positively to years of education (Li et al., 2013), and to be worse in male participants (A. E. Thompson & Voyer, 2014), in non-native speakers (Ishikawa, 2006), and inattentive participants.

**Materials and Methods**

**Participants**

The study was approved by our department’s ethics committee (proposal number 2020-01). Between mid-February 2020 and mid-April 2020 *N* = 584 individuals were recruited by different means of on- and offline advertisement. Native and non-native individuals above the age of 18 could take part in German or in English (*N* = 291 German natives, *N* = 53 German non-natives, *N* = 75 English natives, and *N* = 162 English non-natives). Compensation comprised personalized feedback, a 50 €-raffle per 100 participants, and study participation credit for local psychology undergraduates (*N* = 44). Participants were fairly international, being native to 61 different countries while residing in 37 different countries–though the majority were either German (*N* = 298) or residing in Germany at the time of the study (*N* = 421). Participants identified mostly as females (*N* = 419)**,** were largely in their late twenties (median age = 29, range 18–88), and highly educated (highest degree achieved at a university (*N* = 353)). In summary, the sample was WEIRD (Henrich, Heine, & Norenzayan, 2010a, 2010b).

**Material**

***Self-focus***

Self-focus was measured with the self-focus sentence completion task (SFSC; Exner, 1973) which requires subjects to finish 30 open-ended sentences prompting responses concerning themselves or others, e. g. “If only I could …”. Three raters coded responses across Exner’s four categories: “egocentric” (self-focused), “allocentric” (other-focused), “both”, and “others”. We followed modern research practice and evaluated the SFSC merely based on the egocentric responses but divided by the number of SFSC items (Philippi et al., 2018; Philippi, Dahl, Jany, & Bruce, 2019; Woodruff-Borden et al., 2001). The three raters were psychology students previously trained on a pilot sample (*N* = 73). Interrater reliability was *Fleiss’ κ* = 0.695 (p < 0.001, 95% CI [0.692; 0.697]; (Fleiss & Cohen, 1973).

***Mindfulness***

Trait mindfulness was assessed using the Mindfulness, Attention, and Awareness Scale (MAAS; Brown & Ryan, 2003; Michalak, Heidenreich, Ströhle, & Nachtigall, 2008). The MAAS is a popular 15-item frequency measure of dispositional mindfulness, including receptive awareness of, and attention to what takes place in the present moment (e. g. “I snack without being aware that I am eating.”). All items are reverse-coded and rated on a 6-point Likert scale ranging from “almost always” (1) to “almost never” (6). Measurement reliability was *α* = .84 and *λ_6_* = .84 (Cronbach, 1951; Guttman, 1945).

***ToM***

Here, we used the Double Movie for Assessment of Social Cognition–Multiple Choice (DMASC-MC; Bölte, Fleck, & Dziobek, 2014; Dziobek et al., 2006). Throughout a 15-minute short movie, the DMASC-MC requires participants to answer 44 items on the thoughts, feelings, and intentions of four characters who spend an evening together (e. g. “Why did Michael say that?”). Each time, participants selected from four answers indicating the participant mentalized appropriately, too much, too little, or not at all. We also included five attention checks inquiring which topics have been extensively discussed among the characters (e. g. what to cook for dinner). Our implementation of the DMASC-MC automatically jumped to the next video sequence as soon as participants selected an answer.

***Negative Affect***

Negative trait affect was measured alongside positive affect (not considered here) with the International Positive And Negative Affect Schedule Short Form (I-PANAS-SF; Thompson, 2016) which measures negative trait affect through subjects’ self-rating with five items (‘upset’, ‘hostile’, ‘ashamed’, ‘nervous’, ‘afraid’) on a 5-point Likert scale ranging from “not at all” (1) to “extremely” (5). Measurement reliability was *α* = .79 and *λ_6_* = .77.

***Demographic Information***

Among others, language, language nativity, gender, age, years of education, and ToM task attention were assessed.

**Procedure**

Data collection was done in formr (Arslan & Tata, 2021; Arslan, Walther, & Tata, 2019). After being informed about the purpose of the study and agreeing to its terms and conditions, participants answered to the SFSC, the I-PANAS-SF, the MAAS, the BFI-2-S (not considered here; Rammstedt, Danner, Soto, & John, 2018; Soto & John, 2017), and another pilot questionnaire on the variability in Big Five trait expression (not considered here). Subsequently, participants completed the DMASC-MC and provided demographic information before finishing the study by choosing their means of compensation.

Except for the demographic and compensatory information, responses were mandatory. Due to the estimated length of the study (ca. 1h), participants were invited to take breaks between the tasks. In combination with the lack of a preset study expiration time, this led some individuals to spread their participation over a couple of hours or even days. The median study duration excluding study consent and compensation was 62 min with *N* = 517 subjects participating within 2h, *N* = 56 more participated within 24h, and *N* = 11 taking multiple days up to one week.

**Data Analysis**

***Data Preparation***

We included all participants who got as far as fully completing the DMASC-MC (N = 584) and correctly answered at least 4 out of 5 attention check items during that task (out N = 41). We did not exclude participants for any other reason. Missing data for years of education (N = 48), gender (N = 15), age (N = 10), and language nativity (N = 3) were imputed based on the variables included in the controls model (cf. section: Multiple Regression Analysis) using predictive mean matching for the continuous variables (Landerman, Land, & Pieper, 1997) and logistic regression for categorical variables (Finch, 2010). We deemed a single imputation without variance estimation sufficient because it only concerned control variables.

***Equivalence Testing***

Descriptive statistics include equivalence testing following the *two one-sided test* procedure (TOST; Lakens, 2017; Lakens et al., 2018). This allowed us to judge whether small but according to *null hypothesis significance testing* (NHST) significant differences (*p* < .05) between the German and English subsamples were nevertheless statistically equivalent to zero based on the statistically necessitated threshold of the *smallest reliably detectable effect size* with a 90 % confidence interval.

***Multiple Regression Analysis***

ToM performance was predicted through multiple linear regression. We ran a main effects model of self-focus and mindfulness, an interaction effect model adding the self-focus × mindfulness interaction, and a controls model adding the following control variables: language (English vs German), language nativity (non-native vs native), gender (male vs female), correct attention control items (4 vs. 5), age, years of education, and negative affect. All continuous variables were z-standardized to better meet OLS assumptions, to prevent multicollinearity, and for better comparability across variables (C. Robinson & Schumacker, 2009). For all categorical variables, contrasts were set using weighted effect coding to account for their imbalanced distribution (Te Grotenhuis et al., 2016b).

For the central interaction effect model, we determined the smallest reliably detectable effect size through sensitivity power analysis (given 𝛼 = .05, *power* = .95, *N* = 543) to be Cohen’s *f*^2^ = .03 for the whole model and partial *f*^2^ = .02 for a single predictor (Cohen, 1988). Furthermore, we ran each model as an OLS and an MM-estimated robust regression model (Yohai, 1987) to judge results independent of parametric assumptions.

***Data and Analysis Access and Software***

With exception of the sensitivity power analysis done in G*Power version 3.1 (Faul, Erdfelder, Lang, & Buchner, 2007), data analysis was entirely done in R version 4.0.2 (R. C. Team, 2020) through R Studio version 1.4.1103 (RTeam, 2020) using the following packages: broom (D. Robinson, Hayes, & Couch, 2021), car (Fox & Weisberg, 2019), clickR (Ferrer & Marin, 2020), here (Müller, 2020), interplot (Solt & Hu, 2019), lmtest (Zeileis & Hothorn, 2002), MASS (Venables & Ripley, 2002), mice (Buuren & Groothuis-Oudshoorn, 2011), misty (Yanagida, 2020), performance (Lüdecke, Makowski, Waggoner, & Patil, 2020), psych (Revelle, 2020), sensemakr (Cinelli, Ferwerda, & Hazlett, 2020), sjmisc (Lüdecke, 2018), tidyverse (Wickham et al., 2019), TOSTER (Lakens, 2017), and wec (Te Grotenhuis et al., 2016a).

**Results**

The pseudonymized and scale-aggregated data and the analysis script are publicly available through the Open Science Framework: ([anonymized link](https://osf.io/yneu7/?view_only=b918e2a6f3754411a0079d4f5f1db7e7)). Descriptive statistics for all variables pooled and broken down by participation language are presented in Table 1. The German subsample was slightly more attentive during the ToM task, reported less negative affect, was slightly more self-focused and contained fewer non-native speakers than the English subsample. A correlation matrix is provided in Table 2. Table 3 details the results of the regression analyses. In particular, we provide the OLS and the respective MM-estimated robust counterpart of the main effect model ((F(2;540) = 1.019, *p* = .362, adj. R^2^ < 0.000, Cohen’s *f* ^2^ = .004); (σ^residual^(540) = 0.989, Cohen’s *f* ^2^ = .003)), the interaction effect model ((F(3;539) = 3.124, *p* = .026, adj. R^2^ = .012, Cohen’s *f* ^2^ = .017); (σ^residual^(539) = 0.988, Cohen’s *f* ^2^ = .017)), and the controls model ((F(10;532) = 6.262, p < .001, adj. R^2^ = .088, Cohen’s *f* ^2^ = .118); (σ^residual^(532) = 0.916, Cohen’s *f* ^2^ = .115)). Notably, the self-focus × mindfulness interaction was significant in the OLS and the robust interaction effect models and the controls models. Furthermore, the controls model indicated that age, years of education, language nativity, and ToM task attention were significantly related to ToM performance but did not account for the variance explained by the self-focus × mindfulness interaction. Negative affect and participation language were only significant in the robust model. Lastly, regression diagnostics as well as the near-perfect correlation *r* = .99 between the residuals of the OLS models and their MM-estimated robust counterparts suggest any violations of OLS assumptions were negligible (Tukey, 1991).

|  | | **Total**  *N* = 543 | | **German**  *N* = 334 (61.51 %) | | **English**  *N* = 209 (38.49 %) | | **Statistical**  **Equivalence** | |
| --- | --- | --- | --- | --- | --- | --- | --- | --- | --- |
| **Continuous variables** | | **M** | **SD** | **M** | **SD** | **M** | **SD** | **TOST** | **NHST** |
| ToM performance | | 34.29 | 4.04 | 34.56 | 4.08 | 33.87 | 3.94 | * | ns |
| Age | | 32.19 | 11.57 | 32.80 | 12.87 | 31.22 | 9.05 | * | ns |
| Years of education | | 15.31 | 4.70 | 15.67 | 4.43 | 14.75 | 5.07 | * | * |
| Negative affect | | 2.03 | 0.76 | 1.85 | 0.68 | 2.32 | 0.78 | ns | * |
| Mindfulness | | 3.94 | 0.71 | 3.99 | 0.70 | 3.88 | 0.72 | * | ns |
| Self-focus | | 0.34 | 0.09 | 0.35 | 0.09 | 0.32 | 0.09 | ns | * |
| **Categorical variables** | | ***N*** | **%** | ***N*** | **%** | ***N*** | **%** | **TOST** | **NHST** |
| Nativity | - native | 352 | 64.48 | 286 | 85.63 | 66 | 31.58 | ns | * |
|  | - non-native | 191 | 35.17 | 48 | 14.37 | 143 | 68.42 |  |  |
| Gender | - female | 402 | 74.03 | 254 | 76.05 | 148 | 70.81 | * | ns |
|  | - male | 141 | 25.97 | 80 | 23.95 | 61 | 29.19 |  |  |
| ToM  attention | -5 | 411 | 75.69 | 275 | 82.34 | 136 | 66.51 | ns | * |
|  | -4 | 132 | 24.31 | 59 | 17.66 | 73 | 33.49 |  |  |

**Table 1. Descriptive statistics.** Listed are the pooled and language-group specific (a) means (M) and standard deviations (SD) of the continuous variables and (b) absolute and relative values for the categorical variables after imputation. Furthermore, the overview provides significant (*; at p < .05) and non-significant (ns) null hypothesis significant testing (NHST) and two one-sided test procedure (TOST) results comparing the statistical equivalence of the German and English subsample. A significant NHST result indicates the difference between the German and English subsample were statistically different from zero and a significant TOST result indicates the difference was statistically *equivalent* to zero.

|  | | continuous | | | | | | categorical | | | |
| --- | --- | --- | --- | --- | --- | --- | --- | --- | --- | --- | --- |
|  |  | **ToM**  **performance** | **Age** | **Years of Education** | **Negative Affect** | **Mindfulness** | **Self-focus** | **Language** | **Nativity** | **Gender** | **ToM**  **attention** |
|  | **Age** | -.11 |  |  |  |  |  |  |  |  |  |
|  | **Years of Education** | .13 | .13 |  |  |  |  |  |  |  |  |
|  | **Negative Affect** | -.08 | -.11 | -.03 |  |  |  |  |  |  |  |
|  | **Mindfulness** | -.01 | .18 | .03 | -.32 |  |  |  |  |  |  |
|  | **Self-focus** | .06 | -.16 | .01 | .10 | -.10 |  |  |  |  |  |
| categorical | **Language** | .11 | .08 | .12 | -.39 | .10 | .19 |  |  |  |  |
|  | **Nativity** | .25 | .11 | .07 | -.25 | .03 | .17 | .55 |  |  |  |
|  | **Gender** | -.13 | .24 | -.02 | -.02 | .09 | -.05 | .04 | .00 |  |  |
|  | **ToM attention** | .19 | -.08 | .07 | .07 | .05 | .08 | .19 | .12 | .06 |  |

**Table 2. Correlation matrix.** Continuous–continuous correlations (top left) have been computed as Pearson correlations; categorical–categorical correlations (bottom right) as bias-corrected Cramer’s V; and continuous–categorical correlations (bottom left) as biserial correlations. Note that correlation coefficients cannot be directly compared across combinations of variable types due to different underlying assumptions.

|  | | | **Intercept** | **Self-focus** | **Mindfulness** | **Age** | **Years of**  **Education** | **Negative**  **Affect** | **Gender**  [male] | **Language**  [English] | **Nativity**  [non-native] | **ToM attention**  [4 out of 5] | **Self-focus ×**  **Mindfulness** | **AIC & BIC** |
| --- | --- | --- | --- | --- | --- | --- | --- | --- | --- | --- | --- | --- | --- | --- |
| **Controls Model** | ***f^2*** | | **.02** | .00 | .00 | **.01** | **.02** | .01 | .01 | .01 | **.04** | **.01** | **.01** | 1555 |
|  | ***p*** | | **.002**  ***<.001*** | .315  *.172* | .869  .855 | **.008**  ***.027*** | **.002**  ***<.001*** | .071  ***.024*** | .101  *.235* | .065  ***.034*** | **<.001**  ***<.001*** | **.012**  ***.004*** | **.01**  ***.004*** |  |
|  | **95% CI** | **UL** | **.28**  ***.32*** | .13  *.14* | .08  *.10* | **-.03**  ***-.00*** | **-.21**  ***.23*** | .01  ***-.02*** | .02  *.05* | .25  ***.28*** | **-.25**  ***.19*** | **-.04**  ***-.04*** | **.20**  ***.22*** | 1504 |
|  |  | **LL** | **.06**  ***.01*** | -.04  -.03 | -.09  *.12* | **-.20**  ***-.21*** | **.05**  ***.07*** | -.17  ***-.19*** | -.26  *-.24* | -.01  ***-.01*** | **-.65**  **-.64** | **-.33**  ***-.38*** | **.03**  ***.03*** |  |
|  | **SE** | | **.06**  ***.05*** | .04  *.04* | .04  .04 | **.04**  ***.04*** | **.04**  ***.04*** | .05  ***.05*** | .07  *.07* | .07  ***.07*** | **.10**  ***.10*** | **.07**  ***.07*** | **.04**  ***.04*** |  |
|  | ***β*** | | **.17**  ***.22*** | .04  *.06* | -.01  *-.01* | **-.12**  ***-.10*** | **.13**  ***.14*** | -.08  ***-.10*** | -.12  *-.08* | .12  ***.14*** | **.45**  ***.42*** | **-.19**  ***-.21*** | **.11**  ***.13*** |  |
| **Interaction Effect Model** | ***f^2*** | | .00 | .01 | .00 |  |  |  |  |  |  |  | **.01** | 1562 |
|  | ***p*** | | .778  **Table 3 Regression models.** All continuous variables have been z-standardized and all categorical variables have been weighted effect coded. Note: Significant findings p > .05 are highlighted in bold.  *.073* | .063  *.066* | .921  *.505* |  |  |  |  |  |  |  | **.007**  ***.009*** |  |
|  | **95% CI** | **UL** | .10  *.16* | .17  *.15* | .09  *.13* |  |  |  |  |  |  |  | **.21**  ***.22*** | 1541 |
|  |  | **LL** | -.07  *-.01* | -.00  *-.00* | -.08  *-.08* |  |  |  |  |  |  |  | **.03**  ***.02*** |  |
|  | **SE** | | .04  *.04* | .04  *.04* | .04  *.04* |  |  |  |  |  |  |  | **.05**  ***.05*** |  |
|  | ***β*** | | .01  *.08* | .08  *.08* | .00  *.03* |  |  |  |  |  |  |  | **.12**  ***.12*** |  |
| **Main Effects Model** | ***f^2*** | | .00 | .00 | .00 |  |  |  |  |  |  |  |  | 1563 |
|  | ***p*** | | 1  *.115* | .155  *.164* | .98  *.523* |  |  |  |  |  |  |  |  |  |
|  | **95% CI** | **UL** | .08  *.15* | .15  *.13* | .09  *.13* |  |  |  |  |  |  |  |  | 1546 |
|  |  | **LL** | -.08  *-.02* | -.02  *-.02* | -.08  *-.08* |  |  |  |  |  |  |  |  |  |
|  | **SE** | | .04  *.04* | .04  *.04* | .04  *.04* |  |  |  |  |  |  |  |  |  |
|  | ***β*** | | 0  *.07* | .06  *.06* | .00  *.03* |  |  |  |  |  |  |  |  |  |
|  |  | | OLS  *robust* | OLS  *robust* | OLS  *robust* | OLS  *robust* | OLS  robust | OLS  *robust* | OLS  *robust* | OLS  *robust* | OLS  *robust* | OLS  *robust* | OLS  *robust* | OLS |


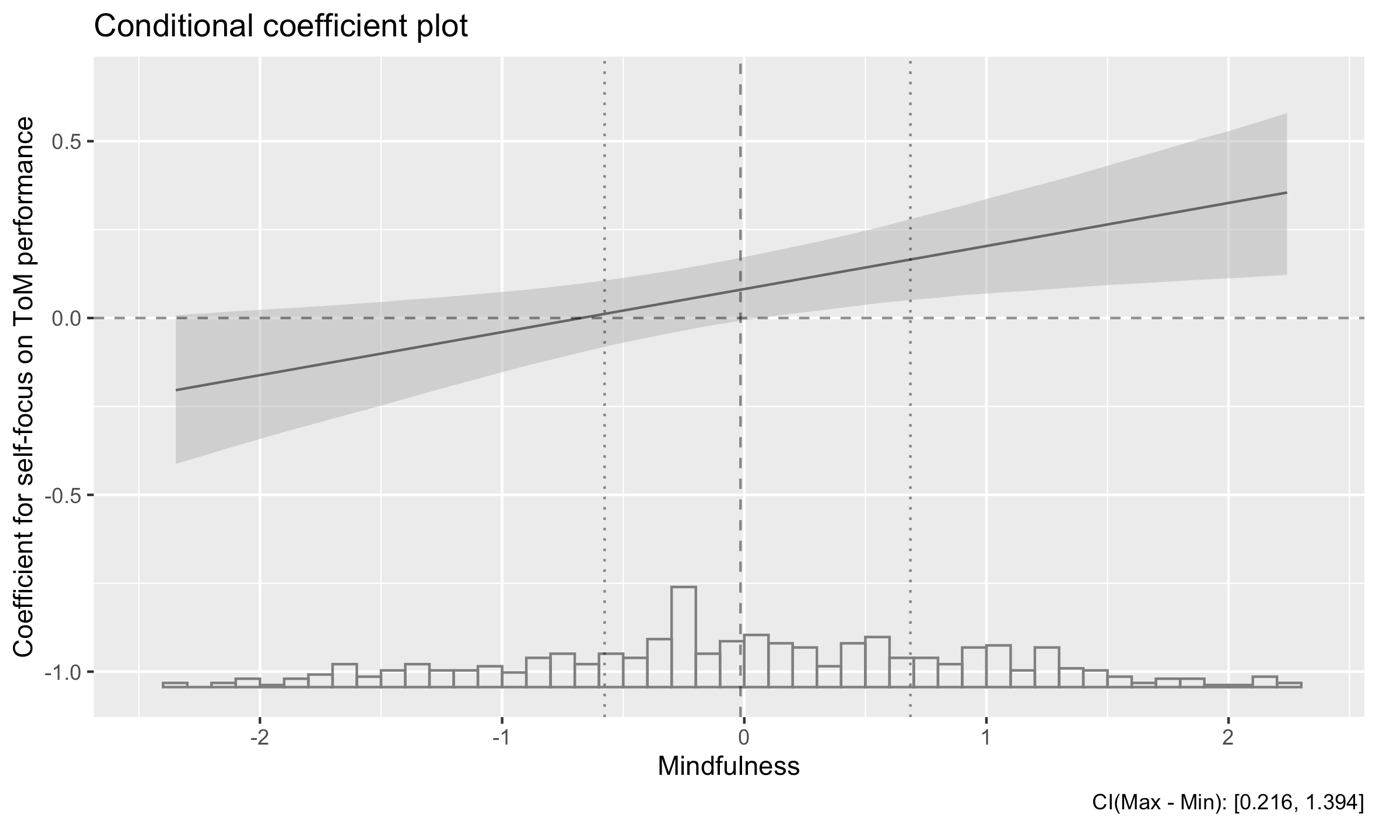


**Figure 1. Conditional coefficient plot** based on the OLS interaction effect model. Dotted vertical lines indicate the interquartile range for mindfulness, the dashed vertical line indicates the median.

**Discussion**

Most importantly, we found support for our central hypothesis (H3) that there is a moderation effect of mindfulness on the relation between self-focus and ToM. The moderation effect was weak but significant in the OLS (*β* = .12, *p* = .007) and the robust (*β* = .12, *p* = .009) interaction model. The relation between self-focus and ToM performance became positive when the median level of mindfulness was exceeded (Figure 1).

Notably, the moderation effect remained about the same strength even after adding control variables to the OLS (*β* = .11, *p* = .01) and the robust model (*β* = .13, *p* = .004). The interaction effect had about the same strength as age (*β* = -.12, *p* = .008) and years of education (*β* = .13, *p* = .002), while attention to the ToM task (*β* = -.19, *p* < .012) was almost twice as strong. Interestingly, language nativity (*β* = -.42, *p* = .03) was still four times as strongly related to ToM performance although participants clearly understood enough about the non-mental content of the conversations in the ToM task to pass the attention check.

A crucial question is whether we should care about the moderation (Götz, Gosling, & Rentfrow, 2021; Lakens et al., 2018; Meyners, 2012) because its effect size was smaller (partia*l* *f* ^2^ = .01) than the smallest reliably detectable effect size as determined by our sensitivity power analysis (*f* ^2^ = .02)–which was coincidentally close to Cohen’s (1988) benchmark for small effects. It suggests that in the long run our finding may be associated with a Type I error rate exceeding the targeted 5% to some extent. By the same reasoning, however, here age and years of education were also unreliable predictors of ToM although their significant relation with ToM has been repeatedly shown. Still, future studies should account for this by increasing their sample size.

Nevertheless, we believe that our results are informative given this study is the first of its kind relating self-focus and ToM accuracy instead of egocentric bias in a sample this large. First, our finding suggests that even if self-focus affects egocentric bias as suggested by previous findings, this may not directly translate into better or worse ToM. A speculative reason may be a “tradeoff of egocentrism” between a person’s own perspective being a source of bias and a source of information when reasoning about other people’s mental states. As argued earlier, bias is usually considered detrimental to accuracy but can be advantageous given noisy information. It may be that mindfully self-focused individuals optimize this trade-off, while absentmindedly self-focused individuals fall short of recognizing their bias or the informativeness of their own perspective.

A second justification for the small but robust moderation effect might be that inferring other people’s minds is a complex task involving a person’s immediate mental state and other situational circumstances like the availability of more target-specific information. From this perspective, even the small interaction effect of two trait-like constructs (self-focus and mindfulness) may seem quite reasonable.

Our first and second hypotheses concerning the positive main effects of self-focus and mindfulness on ToM performance were not supported. However, together with the significant mediation effect, this may only strengthen our main claim that the role of self-focus for ToM is dependent on the quality or content of self-focus like whether self-focus is mindful or absentminded. The finding does not support objective self-awareness theory but it does not directly oppose it either because the SFSC arguably assesses a dispositional form of self-focus, whereas objective self-awareness theory is concerned with the role of state self-focus.

**Limitations**

Previous research on self-focus often relied on (quasi-)experimental designs in smaller samples often inducing different state levels of self-focus while measuring egocentric bias. We analyzed a large cross-sectional dataset including–depending on one’s interpretation of what the SFSC–trait self-focus and a ToM accuracy measure.

The relationships between variables were generally weak with two key measures suffering methodological criticism: although in use for a long time, the SFSC’s validity and reliability are questionable as there has been no formal validation against other measures of self-focus (Exner, 1973) and the MAAS items ask exclusively about absentminded behavior but the absence of absentmindedness might not equal mindfulness (Van Dam, Earleywine, & Borders, 2010). Moreover, our data might have been quite noisy: first, the study’s overall procedure might have been too long and demanding for an online study for which a distraction-free environment cannot be guaranteed; second, collapsing across German and English natives and non-natives might make the observations more heterogeneous without making the findings more generalizable.

**Conclusion**

We hypothesized that the role of self-focus on ToM performance depends on a person’s level of mindfulness so that focusing on oneself, may hinder or facilitate accurate ToM or not. We found a small but robust and significant interaction effect of self-focus and mindfulness according to which there is a positive effect on ToM performance for mindfully self-focused individuals but not for absentminded individuals. Thus, our results provide initial evidence for the idea that ToM performance is differentially influenced by different qualities of self-focus. Future research is needed to investigate the exact mechanisms at work in this relationship.

**References**

Abbate, C. S., Boca, S., & Gendolla, G. H. E. (2016). Self-awareness, Perspective-taking, and Egocentrism. *Http://Dx.Doi.Org/10.1080/15298868.2015.1134638*, *15*(4), 371–380. https://doi.org/10.1080/15298868.2015.1134638

Achim, A. M., Guitton, M., Jackson, P. L., Boutin, A., & Monetta, L. (2013). On what ground do we mentalize? Characteristics of current tasks and sources of information that contribute to mentalizing judgments. *Psychological Assessment*, *25*(1), 117–126. https://doi.org/10.1037/A0029137

Arslan, R. C., & Tata, C. (2021). chain simple forms / surveys into longer runs using the power of R to generate pretty feedback and complex designs https://formr.org. https://doi.org/10.5281/ZENODO.5116576

Arslan, R. C., Walther, M. P., & Tata, C. S. (2019). formr: A study framework allowing for automated feedback generation and complex longitudinal experience-sampling studies using R. *Behavior Research Methods 2019 52:1*, *52*(1), 376–387. https://doi.org/10.3758/S13428-019-01236-Y

Baanders, M. F. (1993). Moods, Social Cognition and Motivation. In H. S. Stam, L. P. Mos, W. Thorngate, & B. Kaplan (Eds.), *Recent Trends in Theoretical Psychology* (pp. 471–477). New Your, NY: Springer, New York, NY. https://doi.org/10.1007/978-1-4612-2746-5_45

Bishop, S. R., Lau, M., Shapiro, S., Carlson, L., Anderson, N. D., Carmody, J., … Devins, G. (2004). Mindfulness: A proposed operational definition. *Clinical Psychology: Science and Practice*, *11*(3), 230–241. https://doi.org/10.1093/CLIPSY.BPH077

Block-Lerner, J., Adair, C., Plumb, J. C., Rhatigan, D. L., & Orsillo, S. M. (2007). The case for mindfulness-based approaches in the cultivation of empathy: Does nonjudgmental, present-moment awareness increase capacity for perspective-taking and empathic concern? *Journal of Marital and Family Therapy*, *33*(4), 501–516. https://doi.org/10.1111/J.1752-0606.2007.00034.X

Bodenhausen, G. V, Sheppard, L. A., & Kramer, G. P. (1994). Negative affect and social judgment: The differential impact of anger and sadness. *European Journal of Social Psychology*, *24*(1), 45–62. https://doi.org/10.1002/EJSP.2420240104

Bölte, S., Fleck, S., & Dziobek, I. (2014). DMASC-MC - Klinisk psykologi - Hogrefe.se. Retrieved from https://hogrefe.se/klinisk-psykologi/dmasc-mc/

Bornemann, B., Herbert, B. M., Mehling, W. E., & Singer, T. (2015). Differential changes in self-reported aspects of interoceptive awareness through 3 months of contemplative training. *Frontiers in Psychology*, *0*(OCT), 1504. https://doi.org/10.3389/FPSYG.2014.01504

Brown, K. W., & Ryan, R. M. (2003). The Benefits of Being Present: Mindfulness and Its Role in Psychological Well-Being. *Journal of Personality and Social Psychology*, *84*(4), 822–848. https://doi.org/10.1037/0022-3514.84.4.822

Buuren, S. van, & Groothuis-Oudshoorn, K. (2011). mice: Multivariate Imputation by Chained Equations in R. *Journal of Statistical Software*, *45*(1), 1–67. https://doi.org/10.18637/JSS.V045.I03

Campos, D., Modrego-Alarcón, M., López-del-Hoyo, Y., González-Panzano, M., Van Gordon, W., Shonin, E., … García-Campayo, J. (2019). Exploring the Role of Meditation and Dispositional Mindfulness on Social Cognition Domains: A Controlled Study. *Frontiers in Psychology*, *0*(APR), 809. https://doi.org/10.3389/FPSYG.2019.00809

Chiou, W.-B., & Lee, C.-C. (2013). Enactment of one-to-many communication may induce self-focused attention that leads to diminished perspective taking: The case of Facebook. *Judgment and Decision Making*, *8*(3), 372–380.

Cinelli, C., Ferwerda, J., & Hazlett, C. (2020). sensemakr: Sensitivity Analysis Tools for Regression Models. Retrieved from https://cran.r-project.org/package=sensemakr

Clore, G. L., Schiller, A. J., & Shaked, A. (2018). Affect and cognition: three principles. *Current Opinion in Behavioral Sciences*, *19*, 78–82. https://doi.org/10.1016/J.COBEHA.2017.11.010

Cohen, J. (1988). Statistical Power Analysis for the Behavioral Sciences. *Statistical Power Analysis for the Behavioral Sciences*. https://doi.org/10.4324/9780203771587

Cronbach, L. J. (1951). Coefficient alpha and the internal structure of tests. *Psychometrika 1951 16:3*, *16*(3), 297–334. https://doi.org/10.1007/BF02310555

Diener, E., & Emmons, R. A. (1984). The independence of positive and negative affect. *Journal of Personality and Social Psychology*, *47*(5), 1105–1117. https://doi.org/10.1037/0022-3514.47.5.1105

Dinulescu, S., Alvi, T., Rosenfield, D., Sunahara, C. S., Lee, J., & Tabak, B. A. (2020). Self-Referential Processing Predicts Social Cognitive Ability: *Https://Doi.Org/10.1177/1948550620902281*, *12*(1), 99–107. https://doi.org/10.1177/1948550620902281

Doesum, N. J. V., Van Lange, D. A. W., & Van Lange, P. A. M. (2013). Social mindfulness: Skill and will to navigate the social world. *Journal of Personality and Social Psychology*, *105*(1), 86–103. https://doi.org/10.1037/A0032540

Duval, S., & Wicklung, R. A. (1972). *A theory of objective self awareness.* Academic Press. Retrieved from https://psycnet.apa.org/record/1973-26817-000

Dziobek, I., Fleck, S., Kalbe, E., Rogers, K., Hassenstab, J., Brand, M., … Convit, A. (2006). Introducing MASC: A Movie for the Assessment of Social Cognition. *Journal of Autism and Developmental Disorders 2006 36:5*, *36*(5), 623–636. https://doi.org/10.1007/S10803-006-0107-0

Exner, J. P. . (1973). The Self Focus Sentence Completion: A Study of Egocentricity. *Http://Dx.Doi.Org/10.1080/00223891.1973.10119902*, *37*(5), 437–455. https://doi.org/10.1080/00223891.1973.10119902

Faul, F., Erdfelder, E., Lang, A.-G., & Buchner, A. (2007). G*Power 3: A flexible statistical power analysis program for the social, behavioral, and biomedical sciences. *Behavior Research Methods 2007 39:2*, *39*(2), 175–191. https://doi.org/10.3758/BF03193146

Fenigstein, A., & Abrams, D. (1993). Self-Attention and the Egocentric Assumption of Shared Perspectives. *Journal of Experimental Social Psychology*, *29*(4), 287–303. https://doi.org/10.1006/JESP.1993.1013

Ferrer, V. F., & Marin, D. H. (2020). clickR: Fix Data and Create Report Tables from Different Objects. Retrieved from https://cran.r-project.org/package=clickR

Finch, W. H. (2010). Imputation Methods for Missing Categorical Questionnaire Data: A Comparison of Approaches. *Journal of Data Science*, *8*(3), 361–378. https://doi.org/10.6339/JDS.2010.08(3).612

Fleiss, J. L., & Cohen, J. (1973). The Equivalence of Weighted Kappa and the Intraclass Correlation Coefficient as Measures of Reliability: *Http://Dx.Doi.Org/10.1177/001316447303300309*, *33*(3), 613–619. https://doi.org/10.1177/001316447303300309

Forgas, J. P. (2002). Feeling and thinking: The influence of affect on social cognition and behavior. In Hofsten (Ed.), *Psychology at the turn of the millennium, Vol. 1. Cognitive, biological, and health perspectives* (pp. 455–480). UK: Psychology Press/Taylor & Francis. Retrieved from https://psycnet.apa.org/record/2004-00053-020

Fox, J., & Weisberg, S. (2019). CAR - An R Companion to Applied Regression. *Thousand Oaks CA: Sage.*, (September 2012), 2016. Retrieved from http://socserv.socsci.mcmaster.ca/jfox/Books/Companion

Frith, C., & Frith, U. (2005). Theory of mind. *Current Biology*, *15*(17), R644–R645. https://doi.org/10.1016/J.CUB.2005.08.041

Fuochi, G., & Voci, A. (2020). A deeper look at the relationship between dispositional mindfulness and empathy: Meditation experience as a moderator and dereification processes as mediators. *Personality and Individual Differences*, *165*, 110122. https://doi.org/10.1016/J.PAID.2020.110122

Furr, R. M. (2008). A Framework for Profile Similarity: Integrating Similarity, Normativeness, and Distinctiveness. *Journal of Personality*, *76*(5), 1267–1316. https://doi.org/10.1111/J.1467-6494.2008.00521.X

Gendolla, G. H. E., & Wicklund, R. A. (2009). Self-Focused Attention, Perspective-Taking, and False Consensus. *Http://Dx.Doi.Org/10.1027/1864-9335.40.2.66*, *40*(2), 66–72. https://doi.org/10.1027/1864-9335.40.2.66

Gigerenzer, G., & Brighton, H. (2009). Homo Heuristicus: Why Biased Minds Make Better Inferences. *Topics in Cognitive Science*, *1*(1), 107–143. https://doi.org/10.1111/J.1756-8765.2008.01006.X

Gigerenzer, G., & Gaissmaier, W. (2010). Heuristic Decision Making. *Http://Dx.Doi.Org/10.1146/Annurev-Psych-120709-145346*, *62*, 451–482. https://doi.org/10.1146/ANNUREV-PSYCH-120709-145346

Golubickis, M., Tan, L. B. G., Falben, J. K., & Macrae, C. N. (2016). The observing self: Diminishing egocentrism through brief mindfulness meditation. *European Journal of Social Psychology*, *46*(4), 521–527. https://doi.org/10.1002/EJSP.2186

Götz, F. M., Gosling, S. D., & Rentfrow, P. J. (2021). Small Effects: The Indispensable Foundation for a Cumulative Psychological Science: *Https://Doi.Org/10.1177/1745691620984483*. https://doi.org/10.1177/1745691620984483

Green, J. D., & Sedikides, C. (2016). Affect and Self-Focused Attention Revisited: The Role of Affect Orientation: *Http://Dx.Doi.Org/10.1177/0146167299025001009*, *25*(1), 104–119. https://doi.org/10.1177/0146167299025001009

Guttman, L. (1945). A basis for analyzing test-retest reliability. *Psychometrika 1945 10:4*, *10*(4), 255–282. https://doi.org/10.1007/BF02288892

Harvey, A., Watkins, E., Mansell, W., & Shafran, R. (2004). *Cognitive Behavioural Processes across Psychological Disorders*. *Cognitive Behavioural Processes across Psychological Disorders*. Oxford University Press. https://doi.org/10.1093/med:psych/9780198528883.001.0001

Hass, R. G., & Eisenstadt, D. (1990). The effects of self-focused attention on perspective-taking and anxiety. *Http://Dx.Doi.Org/10.1080/08917779008249334*, 55–66. https://doi.org/10.1080/08917779008249334

Henrich, J., Heine, S. J., & Norenzayan, A. (2010a). Most people are not WEIRD. *Nature 2010 466:7302*, *466*(7302), 29–29. https://doi.org/10.1038/466029a

Henrich, J., Heine, S. J., & Norenzayan, A. (2010b). The weirdest people in the world? *Behavioral and Brain Sciences*, *33*(2–3), 61–83. https://doi.org/10.1017/S0140525X0999152X

Henry, J. D., Phillips, L. H., Ruffman, T., & Bailey, P. E. (2013). A meta-analytic review of age differences in theory of mind. *Psychology and Aging*, *28*(3), 826–839. https://doi.org/10.1037/A0030677

Huffziger, S., & Kuehner, C. (2009). Rumination, distraction, and mindful self-focus in depressed patients. *Behaviour Research and Therapy*, *47*(3), 224–230. https://doi.org/10.1016/J.BRAT.2008.12.005

Ingram, R. E. (1990). Self-focused attention in clinical disorders: Review and a conceptual model. *Psychological Bulletin*, *107*(2), 156–176. Retrieved from https://psycnet.apa.org/buy/1990-14799-001

Ishikawa, T. (2006). The Effect of Task Complexity and Language Proficiency on Task-Based Language Performance. *THE JOURNAL OF ASIA TEFL*, *3*(4), 193–225.

Kingsbury, E. (2009). The relationship between empathy and mindfulness: Understanding the role of self-compassion. - PsycNET. *Dissertation Abstracts International: Section B: The Sciences and Engineering*, *70*(5-B), 3175. Retrieved from https://psycnet.apa.org/record/2009-99220-292

Lakens, D. (2017). Equivalence Tests: A Practical Primer for t Tests, Correlations, and Meta-Analyses. *Http://Dx.Doi.Org/10.1177/1948550617697177*, *8*(4), 355–362. https://doi.org/10.1177/1948550617697177

Lakens, D., Scheel, A. M., & Isager, P. M. (2018). Equivalence Testing for Psychological Research: A Tutorial: *Https://Doi.Org/10.1177/2515245918770963*, *1*(2), 259–269. https://doi.org/10.1177/2515245918770963

Landerman, L. R., Land, K. C., & Pieper, C. F. (1997). An Empirical Evaluation of the Predictive Mean Matching Method for Imputing Missing Values: *Http://Dx.Doi.Org/10.1177/0049124197026001001*, *26*(1), 3–33. https://doi.org/10.1177/0049124197026001001

Li, X., Wang, K., Wang, F., Tao, Q., Xie, Y., & Cheng, Q. (2013). Aging of theory of mind: The influence of educational level and cognitive processing. *International Journal of Psychology*, *48*(4), 715–727. https://doi.org/10.1080/00207594.2012.673724

Lüdecke, D. (2018). sjmisc: Data and Variable Transformation Functions. *Journal of Open Source Software*, *3*(26), 754. https://doi.org/10.21105/JOSS.00754

Lüdecke, D., Makowski, D., Waggoner, P., & Patil, I. (2020). performance: Assessment of Regression Models Performance. *CRAN*. https://doi.org/10.5281/zenodo.3952174

McKie, A., Askew, K., & Dudley, R. (2017). An experimental investigation into the role of ruminative and mindful self-focus in non-clinical paranoia. *Journal of Behavior Therapy and Experimental Psychiatry*, *54*, 170–177. https://doi.org/10.1016/J.JBTEP.2016.07.014

Meyners, M. (2012). Equivalence tests – A review. *Food Quality and Preference*, *26*(2), 231–245. https://doi.org/10.1016/J.FOODQUAL.2012.05.003

Michalak, J., Heidenreich, T., Ströhle, G., & Nachtigall, C. (2008). Die deutsche Version der Mindful Attention and Awareness Scale (MAAS) Psychometrische Befunde zu einem Achtsamkeitsfragebogen. *Http://Dx.Doi.Org/10.1026/1616-3443.37.3.200*, *37*(3), 200–208. https://doi.org/10.1026/1616-3443.37.3.200

Mor, N., & Winquist, J. (2002). Self-focused attention and negative affect: A meta-analysis. *Psychological Bulletin*, *128*(4), 638–662. https://doi.org/10.1037/0033-2909.128.4.638

Müller, K. (2020). A Simpler Way to Find Your Files [R package here version 1.0.1]. Retrieved from https://cran.r-project.org/package=here

Panayiotou, G. (2004). Performance effects of self-focused attention among evaluatively anxious and normal individuals: A review of the literature. Psychology. *The Journal of the Hellenic Psychological Society*, *11*(2), 255–268. Retrieved from https://gnosis.library.ucy.ac.cy/handle/7/37512

Philippi, C. L., Cornejo, M. D., Frost, C. P., Walsh, E. C., Hoks, R. M., Birn, R., & Abercrombie, H. C. (2018). Neural and behavioral correlates of negative self-focused thought associated with depression. *Human Brain Mapping*, *39*(5), 2246–2257. https://doi.org/10.1002/HBM.24003

Philippi, C. L., Dahl, G., Jany, M., & Bruce, S. E. (2019). Impact Statement Coding of Self-Related Thought in Women With Posttraumatic Stress Disorder. *Journal of Traumatic Stress*, *32*(2), 269–276. https://doi.org/10.1002/JTS.22386

Preckel, K., Kanske, P., & Singer, T. (2018). On the interaction of social affect and cognition: empathy, compassion and theory of mind. *Current Opinion in Behavioral Sciences*, *19*, 1–6. https://doi.org/10.1016/J.COBEHA.2017.07.010

Raglan, G. B., & Schulkin, J. (2014). Decision Making, Mindfulness, and Mood: How Mindfulness Techniques can Reduce the Impact of Biases and Heuristics through Improved Decision Making and Positive Affect. https://doi.org/10.4172/2167-1044.1000168

Rammstedt, B., Danner, D., Soto, C. J., & John, O. P. (2018). Validation of the Short and Extra-Short Forms of the Big Five Inventory-2 (BFI-2) and Their German Adaptations. *Https://Doi.Org/10.1027/1015-5759/A000481*, *36*(1), 149–161. https://doi.org/10.1027/1015-5759/A000481

Revelle, W. (2020). psych: Procedures for Psychological, Psychometric, and Personality Research. Evanston, Illinois. Retrieved from https://cran.r-project.org/package=psych

Robinson, C., & Schumacker, R. E. (2009). Interaction Effects: Centering, Variance Inflation Factor, and Interpretation Issues. *Multiple Linear Regression Viewpoints*, *35*(1).

Robinson, D., Hayes, A., & Couch, S. (2021). broom: Convert Statistical Objects into Tidy Tibbles. Retrieved from https://cran.r-project.org/package=broom

Rogers, T. B., Kuiper, N. A., & Kirker, W. S. (1977). Self-reference and the encoding of personal information. *Journal of Personality and Social Psychology*, *35*(9), 677–688. https://doi.org/10.1037/0022-3514.35.9.677

Ross, L., Greene, D., & House, P. (1977). The “false consensus effect”: An egocentric bias in social perception and attribution processes. *Journal of Experimental Social Psychology*, *13*(3), 279–301. https://doi.org/10.1016/0022-1031(77)90049-X

Ross, M., & Sicoly, F. (1979). Egocentric biases in availability and attribution. *Journal of Personality and Social Psychology*, *37*(3), 322–336. https://doi.org/10.1037/0022-3514.37.3.322

Sauer, S. E., & Baer, R. A. (2012). Ruminative and mindful self-focused attention in borderline personality disorder. *Personality Disorders: Theory, Research, and Treatment*, *3*(4), 433–441. https://doi.org/10.1037/A0025465

Schimmack, U., Oishi, S., Diener, E., & Suh, E. (2016). Facets of Affective Experiences: A Framework for Investigations of Trait Affect: *Http://Dx.Doi.Org/10.1177/0146167200268002*, *26*(6), 655–668. https://doi.org/10.1177/0146167200268002

Schroevers, M. J., & Brandsma, R. (2010). Is learning mindfulness associated with improved affect after mindfulness-based cognitive therapy? *British Journal of Psychology*, *101*(1), 95–107. https://doi.org/10.1348/000712609X424195

Silvia, P. J., & Duval, T. S. (2001). Objective Self-Awareness Theory: Recent Progress and Enduring Problems: *Http://Dx.Doi.Org/10.1207/S15327957PSPR0503_4*, *5*(3), 230–241. https://doi.org/10.1207/S15327957PSPR0503_4

Solt, F., & Hu, Y. (2019). interplot: Plot the Effects of Variables in Interaction Terms. Retrieved from https://cran.r-project.org/package=interplot

Soto, C. J., & John, O. P. (2017). Short and extra-short forms of the Big Five Inventory–2: The BFI-2-S and BFI-2-XS. *Journal of Research in Personality*, *68*, 69–81. https://doi.org/10.1016/J.JRP.2017.02.004

Spector, P. E., & Brannick, M. T. (2010). Methodological Urban Legends: The Misuse of Statistical Control Variables: *Https://Doi.Org/10.1177/1094428110369842*, *14*(2), 287–305. https://doi.org/10.1177/1094428110369842

Steinbeis, N. (2016). The role of self–other distinction in understanding others’ mental and emotional states: neurocognitive mechanisms in children and adults. *Philosophical Transactions of the Royal Society B: Biological Sciences*, *371*(1686). https://doi.org/10.1098/RSTB.2015.0074

Stephenson, B., & Wicklund, R. A. (1983). Self-directed attention and taking the other’s (https://doi.org/10.1016/0022-1031(83)90005-7

Te Grotenhuis, M., Pelzer, B., Eisinga, R., Nieuwenhuis, R., Schmidt-Catran, A., & Konig, R. (2016a). When size matters: advantages of weighted effect coding in observational studies. *International Journal of Public Health 2016 62:1*, *62*(1), 163–167. https://doi.org/10.1007/S00038-016-0901-1

Te Grotenhuis, M., Pelzer, B., Eisinga, R., Nieuwenhuis, R., SChmidt-Catran, A., & Konig, R. (2016b). A novel method for modelling interaction between categorical variables. *International Journal of Public Health*, 1–5. Retrieved from http://doi.org/10.1007/s00038-016-0902-0

Team, R. C. (2020). R: The R Project for Statistical Computing. Retrieved August 6, 2021, from https://www.r-project.org/

Team, Rs. (2020). RStudio | Open source & professional software for data science teams - RStudio. Retrieved August 6, 2021, from https://www.rstudio.com/

Thompson, A. E., & Voyer, D. (2014). Sex differences in the ability to recognise non-verbal displays of emotion: A meta-analysis. *Https://Doi.Org/10.1080/02699931.2013.875889*, *28*(7), 1164–1195. https://doi.org/10.1080/02699931.2013.875889

Thompson, E. R. (2016). Development and Validation of an Internationally Reliable Short-Form of the Positive and Negative Affect Schedule (PANAS): *Http://Dx.Doi.Org/10.1177/0022022106297301*, *38*(2), 227–242. https://doi.org/10.1177/0022022106297301

Todd, A. R., Forstmann, M., Burgmer, P., Brooks, A. W., & Galinsky, A. D. (2015). Anxious and egocentric: How specific emotions influence perspective taking. *Journal of Experimental Psychology: General*, *144*(2), 374–391. https://doi.org/10.1037/XGE0000048

Trapnell, P. D., & Campbell, J. D. (1999). Private self-consciousness and the five-factor model of personality: Distinguishing rumination from reflection. *Journal of Personality and Social Psychology*, *76*(2), 284–304. https://doi.org/10.1037/0022-3514.76.2.284

Tukey, J. W. (1991). The Philosophy of Multiple Comparisons. *Statistical Science*, *6*(1), 100–116. Retrieved from https://www.jstor.org/stable/2245714?seq=1#metadata_info_tab_contents

Van Dam, N. T., Earleywine, M., & Borders, A. (2010). Measuring mindfulness? An Item Response Theory analysis of the Mindful Attention Awareness Scale. *Personality and Individual Differences*, *49*(7), 805–810. https://doi.org/10.1016/J.PAID.2010.07.020

Venables, W. N., & Ripley, B. D. (2002). *Modern Applied Statistics with S* (Fourth). New York: Springer. Retrieved from http://www.stats.ox.ac.uk/pub/MASS4/

Watkins, E. R. (2008). Constructive and unconstructive repetitive thought. *Psychological Bulletin*, *134*(2), 163. https://doi.org/10.1037/0033-2909.134.2.163

Watkins, E., & Teasdale, J. D. (2004). Adaptive and maladaptive self-focus in depression. *Journal of Affective Disorders*, *82*(1), 1–8. https://doi.org/10.1016/J.JAD.2003.10.006

Watson, D., Clark, L. A., & Tellegen, A. (1988). Development and Validation of Brief Measures of Positive and Negative Affect: The PANAS Scales. *Journal of Personality and Social Psychology*, *54*(6), 1063–1070. https://doi.org/10.1037/0022-3514.54.6.1063

Weger, U. W., Hooper, N., Meier, B. P., & Hopthrow, T. (2012). Mindful maths: Reducing the impact of stereotype threat through a mindfulness exercise. *Consciousness and Cognition*, *21*(1), 471–475. https://doi.org/10.1016/J.CONCOG.2011.10.011

Wickham, H., Averick, M., Bryan, J., Chang, W., McGowan, L. D., François, R., … Yutani, H. (2019). Welcome to the Tidyverse. *Journal of Open Source Software*, *4*(43), 1686. https://doi.org/10.21105/JOSS.01686

Woodruff-Borden, J., Brothers, A. J., & Lister, S. C. (2001). SELF-FOCUSED ATTENTION: COMMONALITIES ACROSS PSYCHOPATHOLOGIES AND PREDICTORS. *Behavioural and Cognitive Psychotherapy*, *29*(2), 169–178. https://doi.org/10.1017/S1352465801002041

Yanagida, T. (2020). misty: Miscellaneous Functions “T. Yanagida.” Retrieved from https://cran.r-project.org/package=misty

Yang, M. L., Yang, C. C., & Chiou, W. Bin. (2010). When guilt leads to other orientation and shame leads to egocentric self-focus: Effects of differential priming of negative affects on perspective taking. *Social Behavior and Personality*, *38*(5), 605–614. https://doi.org/10.2224/SBP.2010.38.5.605

Yohai, V. J. (1987). High Breakdown-Point and High Efficiency Robust Estimates for Regression on JSTOR. *The Annals of Statistics*, *15*(20), 642–656. Retrieved from https://www.jstor.org/stable/2241331?seq=1#metadata_info_tab_contents

Zeileis, A., & Hothorn, T. (2002). Diagnostic Checking in Regression Relationships. *R News*, *2*(3), 7–10. Retrieved from https://cran.r-project.org/doc/Rnews/
