# Supplementary figures and images for: Mindful self-focus–an interaction affecting Theory of Mind?
Source: PLoS One. 2023 Feb 2;18(2):e0279544. doi: 10.1371/journal.pone.0279544 (PMC9894420; doi:10.1371/journal.pone.0279544)

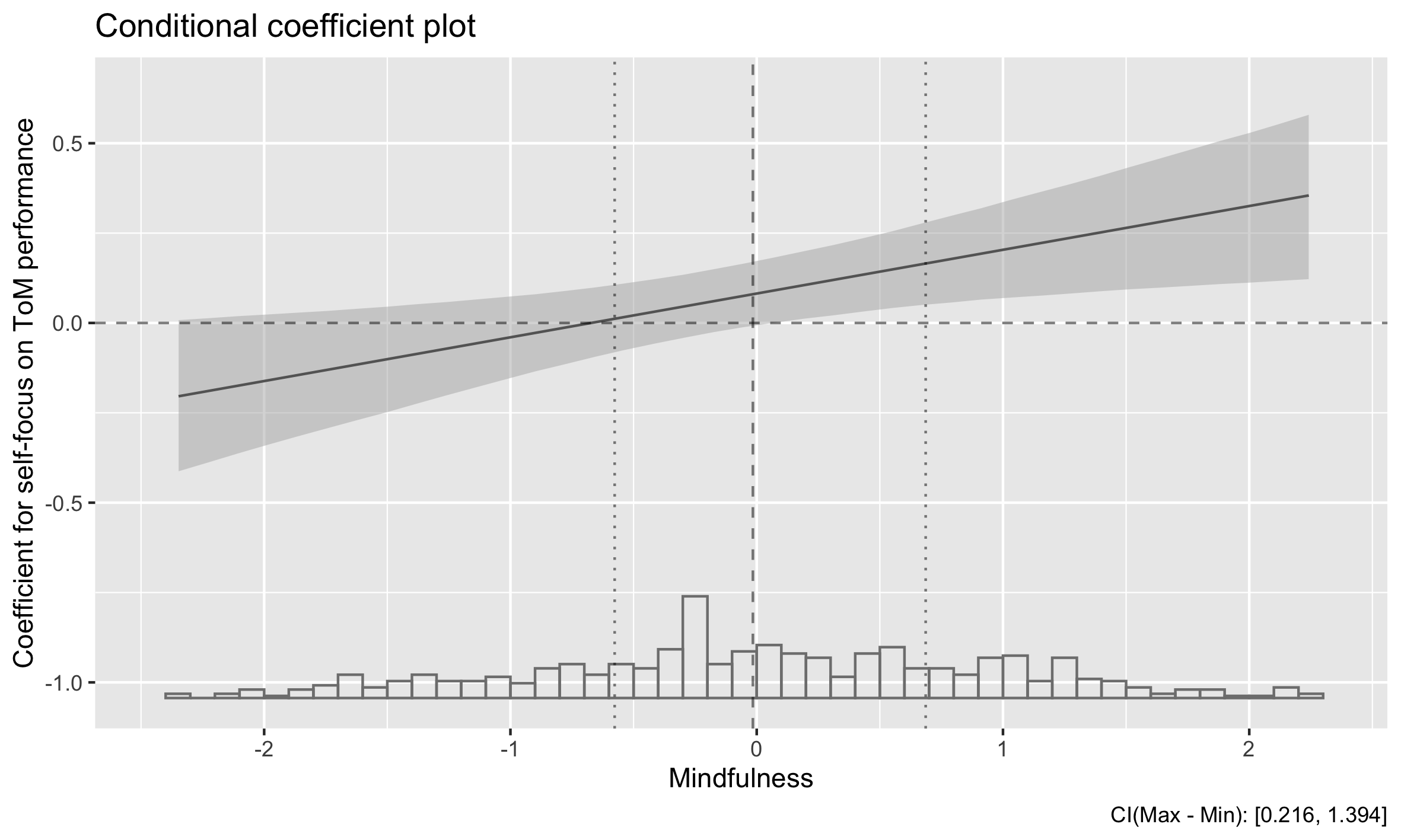

Supplement: S1 File — (ZIP) [file pone.0279544.s001.zip › Processing and Analysis/Analysis Data/interactionCoefficient.png]

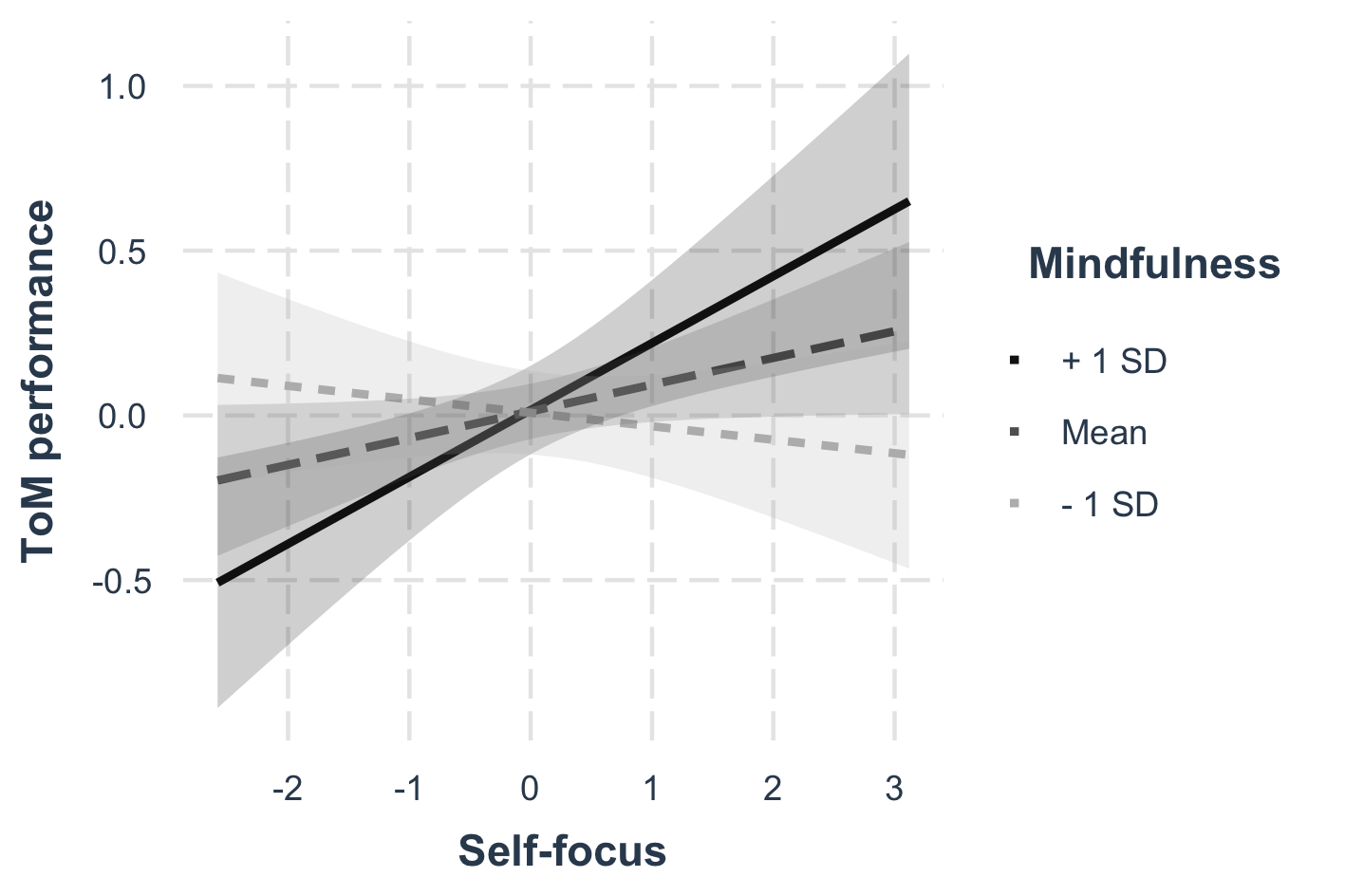

Supplement: S2 File — (ZIP) [file pone.0279544.s002.zip › mindful_selffocus_data&analysisscript/Processing and Analysis/Analysis Data/Manuscript Figures and Tables/fig2_interactionEffectSD.tiff]

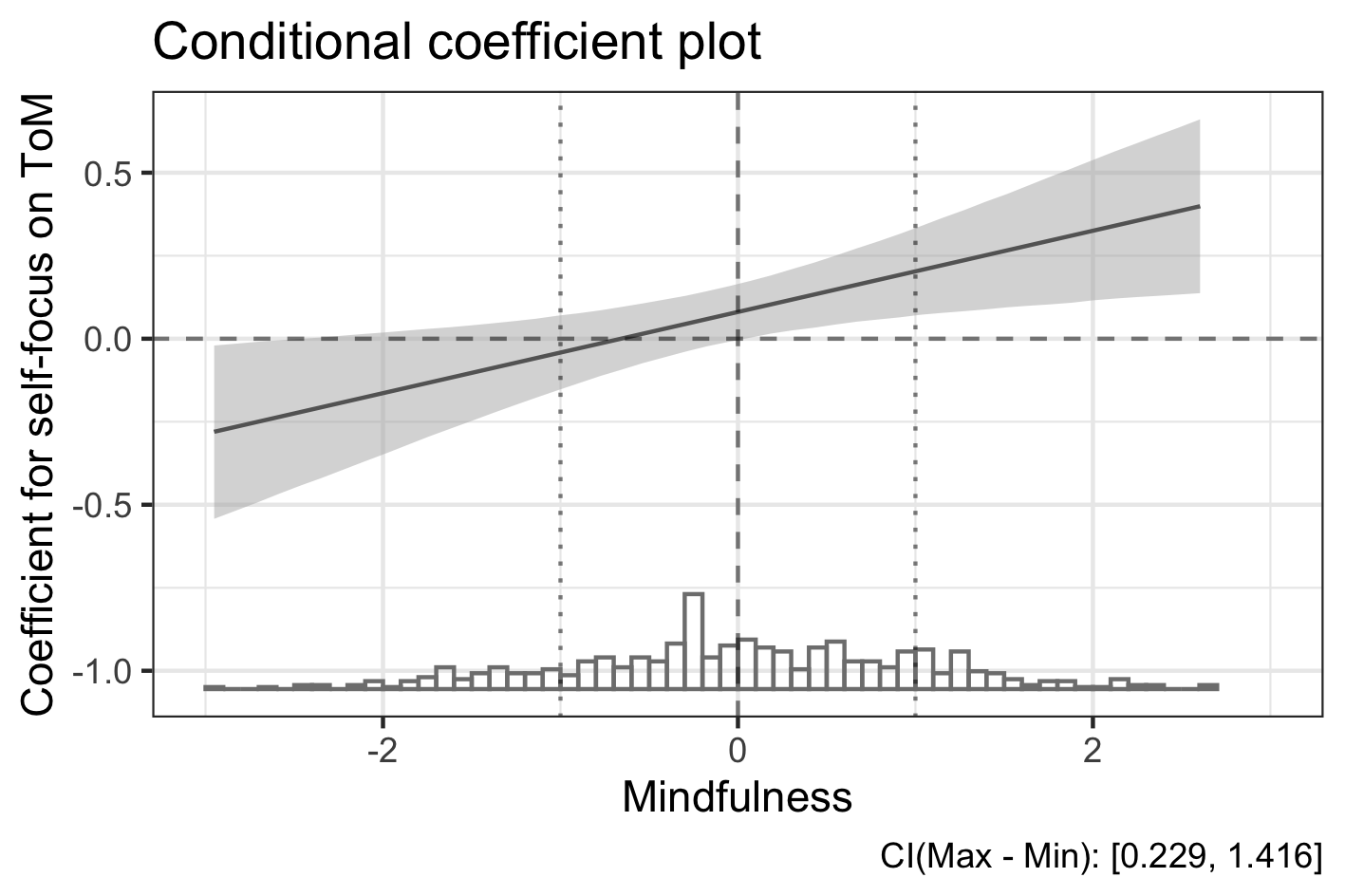

Supplement: S2 File — (ZIP) [file pone.0279544.s002.zip › mindful_selffocus_data&analysisscript/Processing and Analysis/Analysis Data/Manuscript Figures and Tables/fig1_interactionCoefficientSD.tiff]
